# Distinct Uptake Routes Participate in Silver Nanoparticle Engulfment by Earthworm and Human Immune Cells

**DOI:** 10.3390/nano12162818

**Published:** 2022-08-17

**Authors:** Bohdana Kokhanyuk, Viola Bagóné Vántus, Balázs Radnai, Eszter Vámos, Gyula Kajner, Gábor Galbács, Elek Telek, Mária Mészáros, Mária A. Deli, Péter Németh, Péter Engelmann

**Affiliations:** 1 Department of Immunology and Biotechnology, Clinical Center, Medical School, University of Pécs, H-7624 Pécs, Hungary; 2 Department of Biochemistry and Medicinal Chemistry, Medical School, University of Pécs, H-7624 Pécs, Hungary; 3 Department of Inorganic and Analytical Chemistry, Faculty of Science and Informatics, University of Szeged, H-6720 Szeged, Hungary; 4 Department of Biophysics, Medical School, University of Pécs, H-7624 Pécs, Hungary; 5 Institute of Biophysics, Biological Research Centre, Eötvös Loránd Research Network, H-6726 Szeged, Hungary

**Keywords:** AgNPs, coelomocytes, endocytosis, monocytes, phylogenesis

## Abstract

The consequences of engineered silver nanoparticle (AgNP) exposure and cellular interaction with the immune system are poorly understood. The immunocytes of the *Eisenia andrei* earthworm are frequently applied in ecotoxicological studies and possess functional similarity to vertebrate macrophages. Hence, we characterized and compared the endocytosis mechanisms for the uptake of 75 nm AgNPs by earthworm coelomocytes, human THP-1 monocytes, and differentiated THP-1 (macrophage-like) cells. Our results indicate that microtubule-dependent, scavenger–receptor, and PI3K signaling-mediated macropinocytosis are utilized during AgNP engulfment by human THP-1 and differentiated THP-1 cells. However, earthworm coelomocytes employ actin-dependent phagocytosis during AgNPs uptake. In both human and earthworm immunocytes, AgNPs were located in the cytoplasm, within the endo-/lysosomes. We detected that the internalization of AgNPs is TLR/MyD88-dependent, also involving the bactericidal/permeability-increasing protein (BPI) in the case of human immunocytes. The exposure led to decreased mitochondrial respiration in human immunocytes; however, in coelomocytes, it enhanced respiratory parameters. Our findings provide more data about NP trafficking as nano-carriers in the nanomedicine field, as well as contribute to an understanding of the ecotoxicological consequences of nanoparticle exposure.

## 1. Introduction

Over the past few decades, metal nanoparticles (NPs) have drawn many researchers’ attention and offer several industrial and medical applications. Among others, metal NPs are utilized in production as catalysts, sensors, and semiconductors [1]; in wastewater treatment [2]; and, significantly, in drug-delivery systems [3]. Nanosilver (AgNPs) is the most widely applied type of metal NP due to its antimicrobial characteristics [4,5]. Hence, the release of AgNPs into the environment during production and application, and the consequent toxic effects on living organisms, is a subject of investigation and concern [6,7,8,9]. When ending up in the terrestrial environment, AgNPs potentially pose a risk to soil-dwelling organisms [10]. Among these, invertebrates are among the first to encounter AgNPs. Hence, *Eisenia andrei* earthworms, as a soil sentinel organism, are highly applicable for toxicity testing [5]. At the cellular level, the innate immune cells, such as phagocytes, are the “first-responders” to environmental contaminants, including AgNPs. The immune cells of *E. andrei*, so-called coelomocytes, include the amoebocyte and eleocyte subpopulations [11]. The amoebocytes exhibit the typical morphological features of phagocytes, such as phagolysosomal granules and filopodia [12]. Recently, we proved that amoebocytes possess a functionally conserved phagocytic machinery to eliminate microbes in a similar manner to vertebrate phagocytes [13].

While data on the toxicological effects of AgNP exposure are abundant [14], the endocytosis mechanisms involved in AgNP uptake are known mostly from vertebrate cells [15], since restricted data are available about the endocytosis pathways for AgNPs in invertebrate cells [16,17,18,19], and practically no information on these mechanisms is available for earthworms.

Investigating AgNP uptake in earthworm coelomocytes is important from an ecotoxicological perspective, while the endocytosis mechanisms of human immunocytes are critical for the improvement of drug-delivery systems. For example, changing particles’ physico-chemical properties can be used to direct them to a specific pathway and for intracellular trafficking. Hence, understanding NPs’ fate in cells can be used to improve the targeting of nanocarriers.

Therefore, in this work, hypothesizing that earthworm amoebocytes share functional similarities with vertebrate myeloid cells and with the aid of pharmacological endocytosis inhibitors, we fill the gap in the research of the 75 nm AgNPs endocytosis mechanisms and related subcellular consequences in three functionally similar invertebrate and vertebrate immune cell types.

## 2. Materials and Methods

### 2.1. Nanoparticles

Polyvinylpyrrolidone (PVP)-coated spherical 75 nm AgNPs (1 mg/mL, BioPure, Cambridge, MA, USA) were purchased from NanoComposix (San Diego, CA, USA), since they are recommended as OECD benchmark materials for nanotoxicological studies. Physical characterization of AgNPs was performed by transmission electron microscopy (TEM), UV/VIS spectrophotometry, dynamic light scattering (DLS), and zeta-potential measurements. Detailed technical information about the characterization can be found in the Supplementary Material.

### 2.2. Earthworm Husbandry and Coelomocyte Isolations

Adult *Eisenia andrei* (Oligochaeta, Lumbricidae) were kept at room temperature (RT) and fed with manure soil. For depuration, earthworms were placed onto moist paper towels one day before the experiments. Coelomocytes were collected by applying an extrusion buffer, then washed in *Lumbricus* balanced salt solution (LBSS) [20]. Then, coelomocytes were enumerated by dead-cell exclusion with trypan blue dye.

### 2.3. Cell Culture Conditions

Human monocytic leukemia cell line THP-1 (ATCC^®^ TIB-202™) was cultured in RPMI medium supplemented with 10% heat-inactivated fetal bovine serum (FBS, Euroclone, Milan, Italy) and 1% penicillin/streptomycin (100 U/mL penicillin and 100 µg/mL streptomycin, Lonza, Basel, Switzerland) at 37 °C in a humidified 5% CO_2_ atmosphere. In order to obtain macrophage-like cells, the differentiation of THP-1 cells (diff. THP-1 cells) was conducted using 5 ng/mL phorbol 12-myristate-13-acetate (PMA) for 48 h under the same culture conditions as described earlier [21]. Differentiation was verified with light microscopy.

### 2.4. Endocytosis Inhibitor Treatments and In Vitro Exposure Conditions

Prior to AgNP exposure, target cells (1 × 10^6^ cells/mL per condition) were placed onto 24-well plates and preincubated with different uptake pathway inhibitors—cytochalasin B, cytochalasin D, colchicine, 5-(N-ethyl-N-isopropyl)amiloride (EIPA), amantadine, methyl-ß-cyclodextrin (MßCD), nystatin, polyinosinic acid (poly(I)), polycytidylic acid (poly(C)), and wortmannin—along with appropriate vehicle controls. All inhibitors were purchased from Sigma-Aldrich (St. Louis, MO, USA). The applied conditions for the inhibitors were chosen based on the literature (please see Table 1).

Following inhibitor pretreatments, THP-1 cells, diff. THP-1 cells, and coelomocytes were further exposed to EC_20_ concentrations of AgNPs (3.1 µg/mL, 3.6 µg/mL, and 38.9 µg/mL, respectively) for 24 h at 37 °C (THP-1 and diff. THP-1) or RT (coelomocytes). Technical details for dose–response curve analysis are provided in the Supplementary Material. During AgNP exposure, we applied RPMI culture medium supplemented with 1% heat-inactivated FBS and 1% penicillin/streptomycin for the three cell types.

### 2.5. Flow Cytometry Measurement of SSC Parameters

For the uptake inhibition experiments, inhibitor-pretreated cells were exposed to the aforementioned EC_20_ doses of AgNPs for 24 h. Upon AgNP uptake, side-scatter (SSC) parameter shifts were measured using a FACSCalibur flow cytometer (Beckton Dickinson, Franklin Lakes, NJ, USA). The intracellular density changes represented by the SSC intensities were analyzed with FCS Express 7.0 software (De Novo Software, Glendale, CA, USA). The derived data are presented as dot plots and flow cytometry histograms.

### 2.6. Transmission Electron Microscopy (TEM) for Intracellular AgNP Localization

Endocytosis inhibitors and/or AgNP-exposed cells were collected, centrifuged, and immersed in 5% glutaraldehyde-phosphate buffer overnight, then in 1% osmium tetroxide in phosphate buffer for 1 h at 4 °C. Next, the washing steps with phosphate buffer and dehydration in an increasing-concentration series of ethanol (50%, 70%, 90%, and 96%) were performed. Then, the samples were placed in propylene oxide, embedded in epoxy resin (Durcupan ACM, Sigma-Aldrich, St. Louis, MO, USA), and stored for 48 h at 56 °C. Samples were mounted on slides and dried overnight at RT. Serial ultrathin sections were cut with a Reichert ultramicrotome and placed on single-slot collodion-coated copper grids (Electron Microscopy Sciences, Fort Washington, PA, USA). According to a standard procedure, the sections were contrasted with uranyl acetate and lead citrate. Samples were examined and images were taken with a JEM-1400 Flash transmission electron microscope (TEM, JEOL, Tokyo, Japan). Fifteen–twenty cells per sample were observed for AgNP localization.

### 2.7. Quantification of Intracellular Silver Content by Inductively Coupled Plasma Mass Spectrometry (ICP-MS)

The exposed samples were collected and centrifuged (5 min at 163 *g*), supernatants were separated, and pellets were resuspended in 1 mL PBS. The cells were enumerated by a LUNA-II automated cell counter (Logos Biosystems, Korea). For ICP-MS measurements, the samples (both pellets and supernatants) were sonicated for 30 sec, vortexed, and used for acidic digestion (ultra-trace quality of 34% HCl and 65% HNO_3_) using hot aqua regia for 1 h at 180 °C. For preparing sample dilutions, trace-quality de-ionized lab water (MilliPoreElix 5 with Synergy, Merck, Kenilworth, NJ, USA) was used. An Agilent 7700× instrument (Agilent, Santa Clara, CA, USA) was calibrated using Ventures IV-ICPMS-71A. During measurements, the signal of the ^107^Ag isotope was monitored. ICP-MS data analysis was performed using Agilent Mass Hunter software (Agilent, Santa Clara, CA, USA).

### 2.8. RNA Isolation, cDNA Synthesis, and Real-Time PCR

The total RNA of the exposed cells was extracted according to the manufacturer’s protocol by applying a NucleoSpin RNA kit (Macherey-Nagel GmbH, Düren, Germany). The quality and quantity of RNA were assessed at 260 nm using a NanoDrop 1000 spectrophotometer (Thermo Fisher Scientific, Waltham, MA, USA). Four independent RNA isolations were performed. The RNA samples were stored at −80 °C. The technical details of cDNA synthesis, real-time PCR conditions, and primer characteristics (Table S1 are included in the Supplementary Material).

### 2.9. Quantification of Global Genomic DNA Methylation Levels

Samples were collected, washed, and prepared for genomic DNA extraction using a NucleoSpin Tissue kit (Macherey-Nagel GmbH) according to the manufacturer’s protocol. The DNA samples were quantified with a NanoDrop 1000 spectrophotometer (Thermo Fisher Scientific) and then stored at −80 °C until use in the Dot blot experiments. The technical details of the 5-methylcytosine Dot blot assay are described in the Supplementary Material. The relative quantification of the dots was performed using ImageLab 6.0 imaging software (Bio-Rad, Hercules, CA, USA). The signals were background-adjusted and the values were normalized to the controls and standard samples.

### 2.10. Bioenergetic Analysis of Mitochondria

Cells were collected and counted as described above. The diff. THP-1 cells were PMA-differentiated directly on XFp cell culture mini plates. A total of 40,000 cells were placed onto the XFp cell culture mini plates and incubated with EC_20_ doses of AgNPs for 12 h, together with vehicle controls; each condition was tested in triplicate. Before measurement, the cell culture medium was replaced with Seahorse XF Assay Media (Agilent, Santa Clara, CA, USA) supplemented with 10 mM of glucose, 2 mM of L-glutamine, and 1 mM of pyruvate. Using a Seahorse XFp Analyzer (Agilent, Santa Clara, CA, USA), a Seahorse XF Cell Mito Stress Test was performed. The oxygen-consumption rate (OCR) and the extracellular acidification rate (ECAR) were measured. For details of the applied inhibitors, explanations of the Seahorse XF Cell Mito Stress Test principles, and details of protein quantification, please see the Supplementary Material. The OCR and ECAR values were normalized to the protein content. The experiments with each cell type were performed at least three times.

### 2.11. Statistical Analysis

The experimental data were analyzed with GraphPad Prism 8.0 software (San Diego, CA, USA) or with Image Lab 6.0 software (Bio-Rad, Hercules, CA, USA). One-way ANOVA with a multiple comparison post hoc test was carried out and the differences were considered significant at *p* < 0.05.

## 3. Results and Discussion

### 3.1. Physico-Chemical Characterization of AgNPs

**Figure 1:**
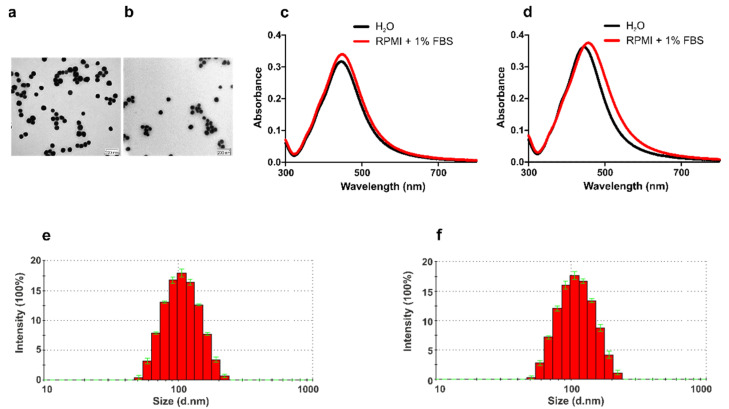
Physico-chemical characterization of AgNPs under exposure conditions. The primary sizes of AgNPs in distilled water (**a**) and RPMI + 1% FBS (**b**) were determined by TEM. Scale bars: 200 nm. UV/VIS spectra (300–800 nm) of AgNPs in distilled water and RPMI + 1% FBS at RT (**c**) and 37 °C (**d**). Histograms from the dynamic light scattering (DLS) measurements demonstrate the hydrodynamic size (nm) and scattering intensity (%) of AgNPs in RPMI + 1% FBS at RT (**e**) and 37 °C (**f**).

Since the physical and chemical properties of NPs are among the major factors that determine NPs’ behavior in biological solutions, it is crucial to analyze and confirm NPs’ statuses in the applied cell culture media before starting NP exposure experiments. To this end, PVP-coated 75 nm AgNPs were resuspended in RPMI supplemented with 1% FBS and 1% penicillin/streptomycin. Subsequently, the primary size was characterized by TEM (Figure 1a,b), and the majority of particles in both solutions was around 75 nm, which is concordant with the manufacturer’s provided information. By means of UV/VIS spectrophotometry, the agglomeration and sedimentation status of AgNPs in different solutions was characterized. Since our experimental conditions required incubation at RT (for coelomocytes) and 37 °C (for human immunocytes), we analyzed the AgNP properties under both temperature conditions (Figure 1c,d).

As a confirmation of particle monodispersity, the light absorbance of the surface plasmon resonance of AgNPs resulted in narrow absorbance peaks. It is worth mentioning the importance of FBS for preventing particle aggregation and their overall stabilization. The proteins in FBS form a biomolecular corona around NPs almost immediately after their introduction into the media. Due to the higher ionic strength of FBS-containing culture media, the formation of protein corona (PC) results in reduced NP aggregation in the solution [32,33]. Therefore, we recorded a slight absorbance peak shift in the sample diluted in RPMI + 1% FBS, a possible consequence of protein adsorption onto the AgNPs’ surface (Figure 1c,d).

DLS measurements (both at RT and 37 °C) were performed in order to evaluate the size and colloidal stability of the AgNPs. We detected a narrow distribution of the particles’ hydrodynamic sizes (Figure 1e,f; Table S2); however, compared with TEM data, the measured mean size values were slightly larger, at approximately 110 nm (RT) and 113 nm (37 °C). This might be explained by the PC formation on the surface of NPs. We observed similar polydispersity under the different temperature conditions, but the ζ potential value was lower at 37 °C. Overall, the results of the physico-chemical characterization of AgNPs concluded that the NPs were in a stable state.

### 3.2. Earthworm and Human Immunocytes Possess Different Sensitivities towards AgNPs

Our major aim was to compare the endocytosis mechanisms of AgNPs in THP-1, diff. THP-1, and coelomocytes. To identify low-cytotoxic concentrations of AgNPs toward the aforementioned cell types, a concentration–response curve analysis was conducted. THP-1 and diff. THP-1 cells were incubated with a 0–10 µg/mL range of AgNP concentrations for 24 h, while we applied a 0–100 µg/mL range of AgNP concentrations to coelomocytes. The cell survival rate was analyzed by flow cytometry following staining with 7-AAD DNA dye. In the case of coelomocytes, it is known that amoebocytes directly participate in AgNP uptake [5]; hence, we concentrated only on the amoebocyte subpopulation during flow cytometry. The concentration–response curves were established (Figure S1) and after normalization, and the following EC_20_ values were calculated: 3.1 µg/mL for THP-1 cells (Figure S1a), 3.6 µg/mL for diff. THP-1 cells (Figure S1b), and 38.9 µg/mL for coelomocytes (Figure S1c).

These results indicate a much higher resistance of earthworm coelomocytes toward AgNPs compared with human immunocytes. This might be due to the fact that earthworms inhabit an environment abundant in chemical elements [34]. Interestingly, our earlier comparative research on THP-1, diff. THP-1 cells, and coelomocytes showed high sensitivity towards AgNPs for all three cell types [5]. These variations might be due to the different commercial sources of NPs (NanoAmor vs. Nanocomposix). These observations emphasize the importance of NP characterization and particles’ physico-chemical properties impact on interactions with cells.

### 3.3. Human THP-1 and Diff. THP-1 Cells Utilize Similar Endocytosis Mechanisms for AgNP Uptake

In the course of evolution, the cells developed various endocytosis mechanisms for the delivery of macromolecules, nutrients, and particles into the cytoplasm. One of the established classifications describes at least two main types of endocytosis, such as phagocytosis and pinocytosis [35]. The last one further includes no less than four types of pinocytosis, such as macropinocytosis, clathrin-mediated endocytosis (CME), caveolae-mediated endocytosis (CvME), and clathrin/caveolin-independent endocytosis. The choice of the particular pathway is defined by the size and nature of the internalized particle, as well as the mechanisms involved in the formation of an endocytic vesicle [35]. A handful of experimental approaches (e.g., knock-down technique and pharmacological compounds) were introduced to study the aforementioned endocytosis pathways [25,35]. In the case of nanoparticle uptake, various pharmacological inhibitors of endocytosis have been successfully applied for invertebrate and vertebrate cells [15,16,17,18,19,36].

**Figure 2:**
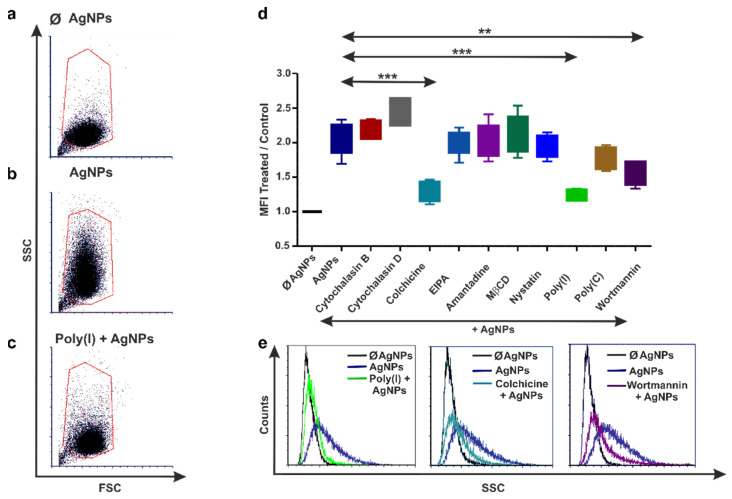
Inhibition of AgNPs’ endocytosis in THP-1 cells. Flow cytometry dot plots (**a**–**c**) showing the side-scatter parameters’ increase after AgNPs’ uptake (**b**) and decrease in cells preincubated with poly(I) (**c**) compared with unexposed (ø AgNP) THP-1 cells (**a**). Graph (**d**) represents the relative efficiency of endocytosis inhibitors on AgNPs’ uptake in THP-1 cells. The boxes show the interquartile ranges (IQR), and the whiskers represent the lowest and highest values of at least four replicates. The statistical significance between different treatments is marked with asterisks (** *p* < 0.01 and *** *p* < 0.001). Representative flow cytometry histograms (**e**) showing the effects of poly(I), colchicine, and wortmannin on AgNPs’ uptake in THP-1 cells.

**Figure 3:**
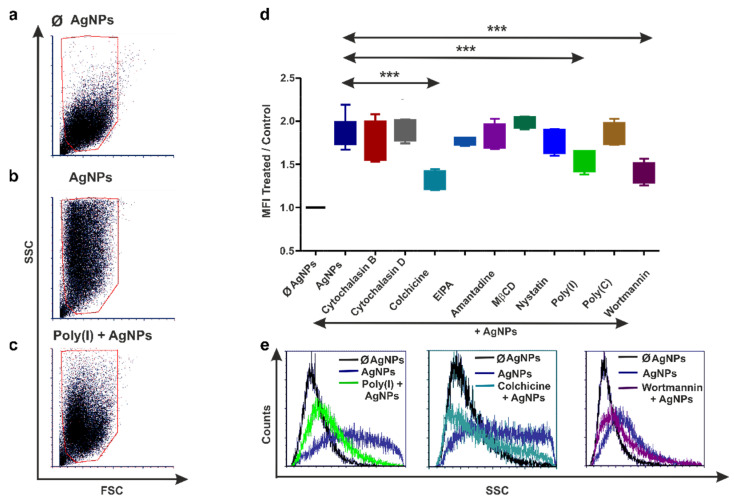
Inhibition of AgNPs’ uptake in diff. THP-1 cells. Representative flow cytometry dot plots (**a**–**c**) demonstrate the increased (**b**) and decreased (**c**) side-scatter parameters upon AgNP exposure or poly(I) preincubation along with AgNP treatments, respectively. Note the side-scatter parameters in the control (ø AgNP) (**a**) diff. THP-1 cells. Graph (**d**) depicts the relative efficiency of different endocytosis inhibitors on AgNPs’ uptake in diff. THP-1 cells. The boxes show the interquartile ranges (IQR), and the whiskers represent the lowest and highest values of at least four replicates. Asterisks mark statistical significance (*** *p* < 0.001) between treatments. Inhibition of AgNPs’ internalization evoked by poly(I), colchicine, and wortmannin is presented on representative flow cytometry histograms (**e**).

**Figure 4:**
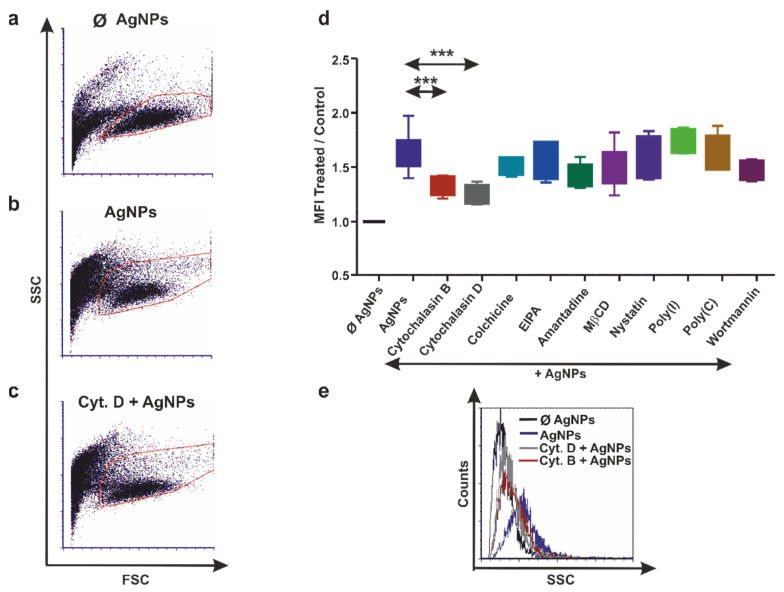
Inhibition of AgNPs' engulfment by coelomocytes. Representative dot plots (**a**–**c**) illustrating the increase in the side-scatter properties of exposed AgNPs (**b**) compared with Ø AgNP control (**a**) coelomocytes. In contrast, the decrease in the side-scatter characteristics was caused by preincubation with cytochalasin D (**c**) in coelomocytes. Graph (**d**) represents the relative efficiency of endocytosis blockers to inhibit AgNPs’ uptake in coelomocytes. The boxes show the interquartile ranges (IQR), and the whiskers represent the lowest and highest values of at least four replicates. Asterisks mark statistical significance (*** *p* < 0.001) between different treatments. Representative flow cytometry histogram (**e**) depicting the inhibition of AgNPs’ uptake by cytochalasin B and cytochalasin D in coelomocytes.

Before applying the pharmacological compounds to block NP uptake, we determined the cytotoxicity of the proposed inhibitor concentrations. The majority (above 75%) of the exposed cell types were alive upon inhibitor treatment (Figure S2a–c). Next, we evaluated the AgNPs’ uptake by THP-1, diff. THP-1 cells, and coelomocytes using flow cytometry assays based on the cells’ SSC properties, a method offered by Suzuki and colleagues [37]. Hence, exposure to NPs, including AgNPs, increases cellular complexity (or granularity), which consequently modulates cells’ SSC. The flow cytometry dot plots illustrate the shift in the SSC parameters upon NPs’ exposure compared with the unexposed (Ø AgNPs) THP-1 (Figure 2a,b), diff. THP-1 cells (Figure 3a,b), and coelomocytes (Figure 4a,b).

The aforementioned flow cytometry principles were applied to identify the efficient pharmacological inhibitors that block the AgNPs’ uptake. Several inhibitors hindered the AgNPs’ engulfment in THP-1 (Figure 2c,e), diff. THP-1 cells (Figure 3c,e), and coelomocytes (Figure 4c,e), as revealed by the SSC parameter changes. Among the ten tested inhibitors, colchicine, poly(I), and wortmannin could attenuate the AgNPs’ uptake in both THP-1 monocytes (Figure 2d,e) and diff. THP-1 cells (Figure 3d,e).

Some of the endocytosis inhibitors in this study interfered with cytoskeleton machinery; for example, cytochalasin B and D blocked actin polymerization, and colchicine arrested microtubules. During endocytosis, actin and microtubules were used as “tracks” for movement; hence, it is essential to understand the cytoskeleton rearrangements in order to evaluate the actions of endocytosis inhibitors during AgNPs’ uptake.

The formation of actin- or microtubule-associated complexes with cargo (e.g., NPs) drives ATP-dependent chemical-to-mechanical energy conversion and the formation of an endocytic vesicle [38]. Then, some vesicles can move on actin first and later use microtubules [39,40].

In the case of phagocytosis, actin filaments are essential for adhesion complexes during cargo binding and phagocytic cup closure. Subsequently, the actin net delivers early phagosomes to the microtubules that continue the endosomal trafficking [41,42,43]. Similarly, for macropinocytosis, at the beginning, myosin associates with macropinocytic cups, but microtubules become responsible when the cups are pinched off from the cell membrane [39,40]. The involvement of actin in other pinocytosis pathways has raised debates among researchers. It has been reported that, in mammalian cells, actin participation is not obligatory; moreover, its involvement is cell-type dependent [44,45,46].

Since, in our study, the actin-depolymerizing agents cytochalasin B and D did not prevent AgNP engulfment by THP-1 and diff. THP-1 cells (Figure 2d and Figure 3d), we can hypothesize that human monocytes and macrophages do not use actin-dependent phagocytosis for AgNPs’ uptake. On the contrary, cells rely on microtubules cytoskeleton machinery, a necessary component of all types of pinocytosis. This is a possible explanation for hindered AgNPs’ internalization after colchicine-mediated microtubule arrest (Figure 2d,e and Figure 3d,e). However, AgNP uptake seems to be cell-type dependent, since the inhibition of 75 nm AgNPs’ uptake in human bronchial epithelial cells was succeeded with cytochalasin D [47].

We also cannot rule out the possibility that THP-1 cells parallelly accomplish several types of pinocytosis as compensatory mechanisms, as well as exhibit different levels of pattern-recognition receptor (PRR) expression for NP engagement. This can be supported by our observations that two additional inhibitors, poly(I) (binds to scavenger receptor (SR) and prevents its association with other ligands) and wortmannin (disrupts phosphoinositide 3-kinase (PI3K) signaling), influence AgNPs’ uptake by THP-1 and diff. THP-1 cells.

Since several classes of SRs are expressed on human myeloid cells, including THP-1 and diff. THP-1 [48], they do not participate in only one specific endocytosis mechanism, but together with ligands (lipoproteins, LPS, engineered NPs, etc.), they can be internalized by CME, CvME, as well as macropinocytosis [49,50,51].

Previously, the involvement of SR was confirmed for ~57 nm and ~45 nm AgNP internalization by murine macrophages [52,53]. In macrophage receptors with collagenous structure (MARCO) SR null murine macrophages, the engulfment of 110 nm large citrate- and PVP-coated AgNPs was compromised [54]. In contrast to our observations, others suggested that the internalization of SR–AgNP complexes occurs through actin-dependent phagocytosis and CME in murine macrophages [52], which emphasizes the species-specific differences between mammalian immunocytes.

As we detected, another necessary component of AgNPs’ uptake is PI3K, and it seems to be required for SR activation in macrophages [55,56], as well as for macropinosomes and early endosome’s fusion during macropinocytosis [41]. Besides an interplay between SR and PI3K signaling, this process also requires microtubules [57]. Previously, the uptake of 80 nm PVP-coated AgNPs by human mesenchymal stem cells also was reduced by wortmannin [15]. Moreover, Lunov et al. [58] suggested that 110 nm polystyrene NPs were also engulfed via the macropinocytosis mechanism in diff. THP-1 cells, but not in THP-1 monocytes.

We also observed that EIPA, an inhibitor of macropinocytosis, did not impact the AgNPs’ uptake, indicating that not only micropinocytosis, but also other clathrin-/caveolin independent pathways, might be involved. Hence, considering all the evidence, we hypothesize that THP-1 and diff. THP-1 cells bind AgNPs through SRs and subsequently internalize them by macropinocytosis as a preferable mechanism involving microtubules, while other endocytosis pathways might work simultaneously and/or interchangeably.

### 3.4. Earthworm Coelomocytes Rely on Actin-Dependent Phagocytosis for AgNPs Uptake

To the best of our knowledge, there are no available data on the endocytosis mechanisms of AgNPs for earthworm coelomocytes. Therefore, the aforementioned endocytosis inhibitors were tested on AgNPs’ uptake in *E. andrei* coelomocytes.

Interestingly, poly(I), colchicine, and wortmannin, which blocked the AgNPs’ uptake by THP-1 and diff. THP-1 cells, did not affect particle internalization by earthworm coelomocytes (Figure 4d). Among the tested inhibitors, cytochalasins B and D blocked AgNPs’ uptake by actin depolymerization, which suggests the leading role of phagocytosis in this process (Figure 4). From the cytoskeletal components, microtubules are involved in AgNP endocytosis by human immunocytes, while earthworm coelomocytes rely on actin filaments. These results may suggest substantial differences in the evolution of endocytosis mediated by invertebrate and vertebrate macrophages.

Regarding endocytosis used by other invertebrate species, the uptake of citrate-coated 25 nm AgNPs by *Caenorhabditis elegans* cells is suggested to involve CME [18]. The uptake of 18 nm AgNPs in *Peringia ulvae* snails is performed by CvME [17]. One pioneering study described the uptake of polymer particles (45 nm) by means of CME and CvME on *Lumbricus rubellus* earthworm cells [59].

Here, we identified that earthworm coelomocytes use actin-dependent phagocytosis for AgNPs’ uptake; however, the exact PRR involved in this process still needs to be identified. Until now, only the *E. andrei* TLR and the lipopolysaccharide-binding protein and bactericidal/permeability-increasing protein (LBP/BPI) have been characterized in detail [60,61]. However, SR is expressed in earthworm tissues [62], and the preincubation of coelomocytes with poly(I) did not confirm SR involvement in AgNPs’ uptake, in contrast to human immunocytes. Notably, our earlier studies of bacterial endocytosis on the same target cells indicated the involvement of a conserved phagocytosis pathway for both invertebrate and vertebrate cells [13]. Hence, it is suggested that earthworm coelomocytes operate with an actin-dependent phagocytosis process in the uptake of both biological (Gram-positive and Gram-negative bacteria) and engineered (AgNP) particles.

### 3.5. AgNPs Do Not Enter the Target Cell Nuclei

**Figure 5:**
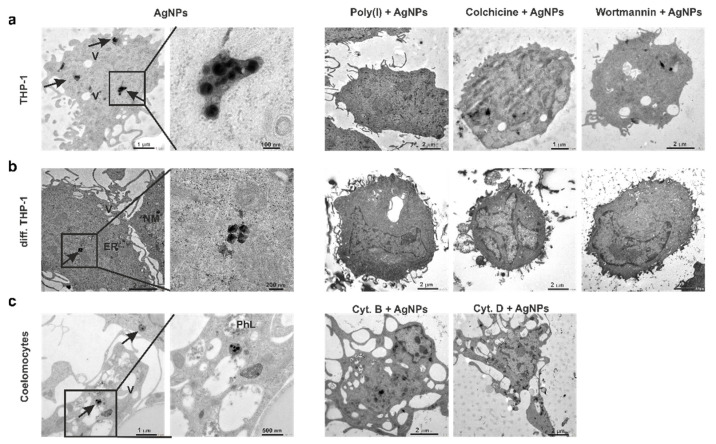
Intracellular localization of AgNPs in THP-1 (**a**), diff. THP-1 (**b**) cells, and coelomocytes (**c**) visualized by TEM. Arrows point to the internalized AgNPs, and letters mark the following organelles: ER—endoplasmic reticulum; NM—nuclear membrane; PhL—phagolysosome; V—vacuoles. Scale bars: 100 nm, 200 nm, 500 nm, 1 μm, and 2 μm.

In order to obtain a visual representation of AgNPs’ intracellular localization and confirm the effect of endocytosis inhibitors, we performed TEM analysis on the exposed target cells. Despite the dissimilar endocytosis pathways used for AgNPs’ uptake (determined by flow cytometry), we did not observe variations in AgNPs’ intracellular localization between human and earthworm immunocytes. TEM images of AgNP-exposed samples evidenced a group of 2–5 NPs in the cytoplasm, close to the cell membrane (Figure 5).

AgNPs were often localized within the endosomes or phagolysosomes. We detected the modified AgNPs’ density inside the diff. THP-1 cells (Figure 5b), which might have been caused by the particles’ dissolution during the 24 h incubation time. Among the earthworm coelomocytes, the amoebocytes subpopulation internalized the majority of the AgNPs, which supports our earlier hypothesis that amoebocytes, but not eleocytes, play an active role in AgNP uptake [5]. Moreover, these observations support the notion that phagocytic amoebocytes are the analog “progenitors” of vertebrate myeloid cells [20,63].

Note that, similar to our earlier studies [5], there were no AgNPs inside the cell nuclei observed in any of the three cell types. The reason might be the size of the nuclear pore channel (25–30 nm), which limits the size of the particles delivered to the nucleus [64]. The particles were also absent in the endoplasmic reticulum and mitochondria.

The TEM images show that the applied pharmacological inhibitors prevented AgNPs’ uptake by the majority of the cells (Figure 5). Hence, after the pre-treatment of THP-1 monocytes with colchicine and further exposure to AgNPs, only 5% of the cell population contained AgNP, 30.7% of cells after poly(I) treatment, and 54.5% of cells that were pre-treated with wortmannin. In case of diff. THP-1 cells, only 24% of the cells engulfed AgNPs after incubation with colchicine, 33.3% cells treated with poly(I), and 28.6% cells treated with wortmannin. For earthworm coelomocytes, these numbers were 46.8% and 21.1% after cytochalasin B and D treatments, respectively (data not shown).

### 3.6. Quantification of Intracellular Silver Content

Inductively coupled mass spectrometry (ICP-MS) was performed in order to detect the amount of silver in the pellets (cells with engulfed NPs) and supernatants (medium with non-internalized silver) to confirm the effects of pharmacological inhibitors on AgNPs’ uptake.

We observed that all three cell types internalized approximately 16–19% of the EC_20_ AgNPs dose, which might indicate the uptake capacity threshold. Similar to the flow cytometry results, THP-1 and diff. THP-1 cells preincubated with poly(I), colchicine, and wortmannin contained less internalized silver compared with the control (ø AgNP) samples. Strikingly, the amount of silver internalized by coelomocytes did not differ between the control and cytochalasins-exposed samples (Table 2).

The reason for this discrepancy might be the fact that the ICP-MS method applied by us does not allow for differentiation between the metal contents in dead/alive cells, nor between the NPs attached to the cell surface or intracellular particles. In turn, the flow cytometry and TEM methods identified changes in cell characteristics before and after particle uptake, and dead cells can be excluded from the analysis. In the flow cytometry measurements of coelomocytes, we could access and evaluate the NPs’ uptake by phagocytic amoebocytes and exclude the non-phagocytic eleocyte subpopulation from analysis, while ICP-MS does not allow distinguishing between the two subtypes. Hence, the variations in the subpopulations’ ratios between the samples could cause discrepancies between the flow cytometry and ICP-MS results that could be resolved by single-particle or single-cell analysis (sp-ICP-MS or sc-ICP-MS, respectively) [65].

### 3.7. TLR/MyD88 Signaling Facilitates AgNPs Internalization

After defining the endocytosis pathways possibly involved in NPs’ uptake, we investigated the downstream signaling mechanism and immune receptor molecules that may be taking part in AgNPs’ endocytic internalization. To maintain a comparative approach, only evolutionary conserved immune response-related genes that were identified in both earthworm and human immunocytes were analyzed, such as *TLR*, *MyD88*, and *BPI* (*LBP/BPI* for earthworms).

**Figure 6:**
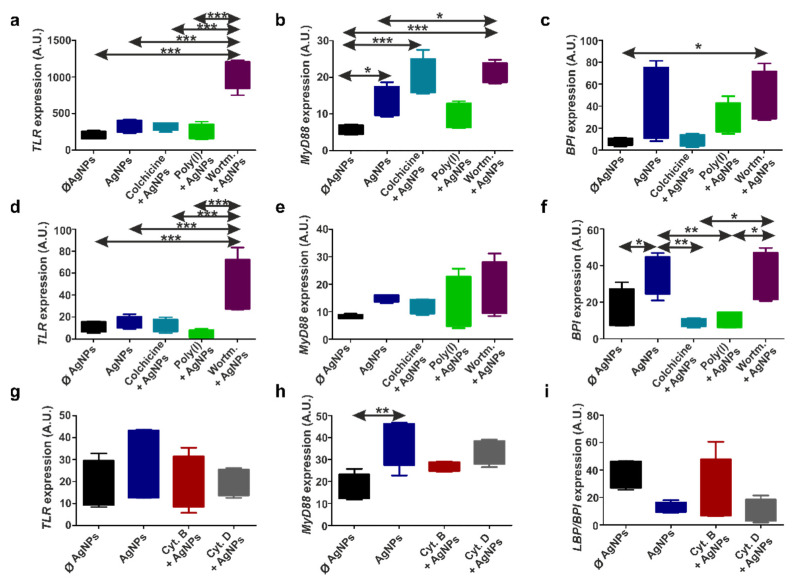
Immune receptor-related gene expression changes caused by endocytosis inhibition and/or AgNP exposure. Graphs represent the normalized expression of *TLR*, *MyD88*, and *BPI* in THP-1 cells (**a**–**c**) and diff. THP-1 cells (**d**–**f**), and normalized expression of *TLR*, *MyD88*, and *LBP*/*BPI* in coelomocytes (**g**–**i**). The boxes represent interquartile ranges (IQR) and whiskers mark the lowest and highest values of four replicates. Asterisks show significantly different samples (* *p* < 0.05, ** *p* < 0.01, and *** *p* < 0.001) analyzed with a one-way ANOVA test. A.U.—arbitrary units.

Similar to earlier results, THP-1 and diff. THP-1 cells showed matching responses to AgNPs on an mRNA expression level. Exposure to AgNPs resulted in a non-significant increase in *TLR* expression (Figure 6a,d).

The preincubation of human immunocytes with colchicine or poly(I) kept *TLR* expression at the control (ø AgNP) level. However, we detected a large wortmannin-induced elevation of *TLR* expression in THP-1 and diff. THP-1 cells (Figure 6a,d). Earlier, similar action on *TLR3* and *TLR4* expression was explained by wortmannin-inhibited PI3K, which is the negative feedback regulator of the TLR cascade. This led to increased NO and IFN-β production, as well as NF-ƙB activation in macrophages and dendritic cells [66,67,68].

Since, in previous experiments, we detected that scavenger receptors take part in AgNPs’ engulfment, it is important to note that, for the initiation of signaling cascades, some scavenger receptors engage with TLRs. In particular, the macrophage scavenger receptor-A (SR-A) associates with TLR4 in response to TLR4 ligands [69], while the class F scavenger receptor expressed by the endothelial cell-I (SREC-I) molecule is also present on the immune cell surfaces and facilitates the membrane translocation of TLR4, leading to the internalization of ligand-TLR4-SREC-I complexes into endosomes [70]. Notably, SREC-I and TLR2 cooperation was reported for invertebrate cells as well [71]. Since we did not detect a strong increase in *TLR* expression, it is possible that TLR was involved at the earlier time points of AgNPs’ uptake. Nevertheless, in agreement with the aforementioned studies, we suggest that SR–TLR complexes are important for AgNPs’ uptake by human immunocytes.

Among several existing classes of SRs and TLRs, it is difficult to define the ones participating in AgNPs’ uptake. For instance, TLR3 also engages with SREC-I and SR-A [72,73], but we excluded the possibility of TLR3 involvement, because TLR3 signaling does not require MyD88, while we observed increased *MyD88* in response to AgNPs by THP-1 and diff. THP-1 cells (Figure 6b,e). In fact, MyD88 serves as an adaptor for all TLR classes, except TLR3 [74]. Earlier, we reported elevated *MyD88* expression by THP-1 cells in response to ~83 nm AgNPs, even after 6 h of exposure [5]. Similar to *TLR* mRNA, the preincubation of THP-1 and diff. THP-1 with wortmannin also increased the *MyD88* levels, indicating PI3K involvement in the MyD88-dependent TLR cascade [75].

To the best of our knowledge, for the first time, we describe here the role of BPI in AgNPs’ uptake by human immunocytes, since there are no data on *BPI* expression upon NP exposure in mammalian cells. Incubation with AgNPs increased the *BPI* expression in THP-1 and diff. THP-1 cells (Figure 6c,f). Preincubation with colchicine and poly(I) caused the downregulation of *BPI* expression after AgNP exposure in THP-1 and diff. THP-1 cells. Similar to the *TLR* levels, the blocking of PI3K signaling by wortmannin caused elevated *BPI* expression, which might be due to the fact that TLR and BPI act as co-receptors responding to the same ligands.

Regarding *E. andrei* earthworm coelomocytes, neither AgNPs nor cytochalasins significantly changed *TLR* expression (Figure 6g). However, similar to human immunocytes, the *MyD88* levels were significantly elevated by AgNP exposure (Figure 6h). In earlier experiments, we already detected high *MyD88* provoked by 50 and 80 nm AgNPs in the closely related earthworm species *Eisenia fetida* [5,76]. It might be possible that *TLR* in coelomocytes was induced earlier than 24 h after AgNPs’ uptake, since the overexpression of *MyD88* that we detected might be a negative regulator of TLR signaling in the late phase of the immune response [77].

Surprisingly, *LBP*/*BPI* did not take part in coelomocytes’ response to AgNPs in our experiments (Figure 6i), since Hayashi et al. [78] previously reported overexpressed *TLR* and *LBP*/*BPI* after *E. fetida* coelomocytes’ exposure to 45 nm AgNPs. Considering these differences, we hypothesize that the responses to AgNPs are *Eisenia* species-specific (as we suggested earlier [79]) and vary depending on the particles’ size and properties. In any case, AgNPs’ uptake might be facilitated by another PRR called coelomic cytolytic factor 1 (CCF-1), which is present in earthworms, but not in humans [80], since previous studies report overexpressed CCF-1 after 24 h of incubation with AgNPs in *E. fetida* [76].

Hence, even though different endocytosis pathways are responsible for AgNPs’ uptake in human and earthworm immunocytes, there are certain similarities in the intracellular immune response to AgNPs [5].

### 3.8. Earthworm Coelomocytes, but Not Human Immunocytes, Exhibit Global DNA Hypermethylation upon AgNPs’ Uptake

The discipline of environmental epigenetics focuses on epigenetic modifications in response to environmental factors, including nanoparticle exposure. While there are already quite abundant data on the mechanisms of environmental epigenetics available from mice and humans, the responses of other organisms, particularly invertebrates, still need to be elucidated [81]. Hence, we aimed to compare the epigenetic (DNA methylation) changes induced by AgNPs and the effects of endocytosis inhibitors in human THP-1 and diff. THP-1 cells, and earthworm coelomocytes.

The genome of *E. andrei* is just partially characterized, and it is difficult to investigate the epigenetic effects of AgNPs on specific genes. Hence, to keep a comparative approach, we explored global DNA methylation changes without the relation to immune response-related genes in human THP-1 monocytes and macrophages, and earthworm coelomocytes.

**Figure 7:**
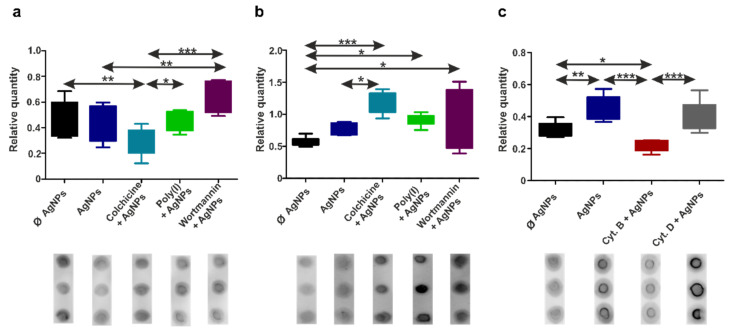
Quantification of 5-mC levels upon endocytosis inhibition and/or AgNP exposure by the Dot blot assay. The graphs represent the relative volumes of 5-mC in the samples seen on the Dot blot images for THP-1 (**a**), diff. THP-1 cells (**b**), and coelomocytes (**c**). The boxes represent the interquartile ranges (IQR), and the whiskers show the lowest and highest values of nine replicates. The statistical significance (* *p* < 0.05, ** *p* < 0.01, and *** *p* < 0.001) between the samples determined by one-way ANOVA test is marked with asterisks. Representative images of the Dot-blot for each condition are provided.

First, we confirmed the specificity of the antibody against 5-methylcytosine (5-mC) using immunocytochemistry (Figure S3), then further assessed the global DNA methylation (5-mCs) pattern by applying the Dot blot technique (Figure 7). Human immunocytes did not exhibit significantly different methylation patterns upon AgNP exposure; however, endocytosis inhibitors caused changes in the cells’ epigenetic profiles (Figure 7a,b). For instance, we detected slight hypermethylation in THP-1 and diff. THP-1 cells caused by wortmannin preincubation, as well as in diff. THP-1 cells after poly(I) preincubation, which suggests increased gene silencing. Preincubation with colchicine, in turn, resulted in decreased 5-mC levels in THP-1 cells (Figure 7a) and increased levels in diff. THP-1 cells (Figure 7b). Similar colchicine-induced hypermethylation was detected earlier for cancer cells [82].

Although AgNPs did not cause 5-mC level changes in THP-1 and diff. THP-1 cells, other epigenetic mechanisms, such as histone modifications and processes involving small and long non-coding RNAs, might be affected. Moreover, the epigenetic response is cell type- and NP type-specific. For example, quantum dots interact with histones, but not DNA or RNA [83], while exposure to CuO NPs results in the hypermethylation of transposable elements in THP-1 cells [84].

In contrast to human immunocytes, we detected hypermethylation in coelomocyte DNA caused by AgNPs’ exposure (Figure 7c). An analogous response to cadmium heavy metal was described in *Lumbricus terrestris* earthworms [85,86], as well as to CuO NPs and nanostructured tungsten carbide cobalt in the soil-dwelling annelid *Enchytraeus crypticus* [87,88]. Similar to human cells, we observed endocytosis inhibitor-related effects, particularly reduced 5-mC levels caused by cytochalasin B (Figure 7c).

Curiously, the conventional invertebrate research models *Drosophila melanogaster* and *Caenorhabditis elegans* possess very weakly detectable changes in DNA methylation; hence, these species are not the best choice for evaluating these variations [89]. In turn, the DNA methylation level in earthworms is relatively high (approx. 13%), which makes earthworms a likely suitable candidate for environmental epigenetics [90].

### 3.9. Cellular Respiration Parameters Changed in Response to AgNPs

In both earthworm and human immunocytes, exposure to AgNPs and their further uptake via the aforementioned endocytosis pathways is an energy-dependent process and a challenge to complete immune and stress responses to NPs. Therefore, the energy metabolism changes involved in AgNPs’ uptake have caught our attention. The mitochondrial stress test was designed to compare the cellular respiration parameters of treated vs. untreated cells; hence, the oxygen consumption rate (OCR) and extracellular acidification rate (ECAR) were measured before and after the incubation of target cells with EC_20_ of AgNP (see Figure S4 for an explanation in the Supplementary Material). In this experimental set-up, we chose a shorter incubation time (12 h) due to additional ROS kinetical analysis (data not shown) compared with the 24 h incubation period.

**Figure 8:**
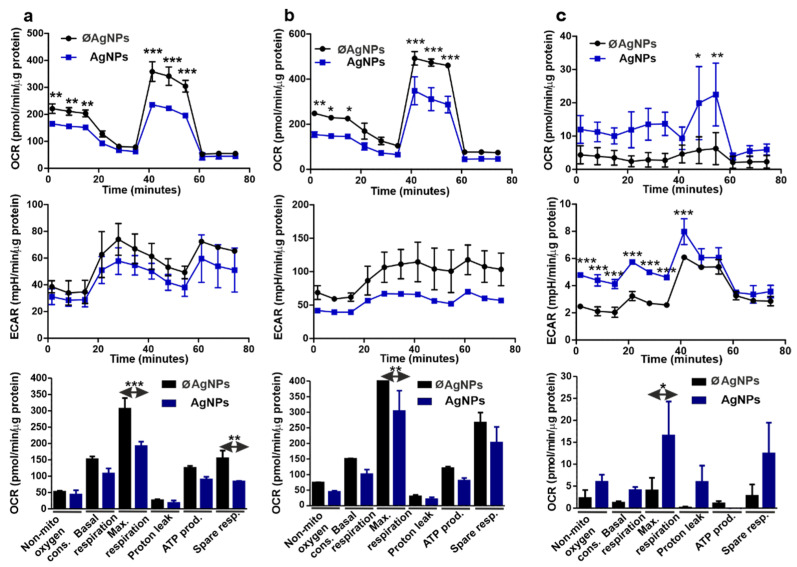
Mitochondrial stress test for the characterization of cell respiratory parameters. The kinetic changes in OCR and ECAR in THP-1 cells (**a**), diff. THP-1 cells (**b**), and coelomocytes (**c**) were measured before and after exposure to AgNPs. The lower set of graphs detail the mitochondrial stress (OCR) parameters. The results were normalized to the protein content. Each experiment was executed at least three times. Asterisks denote statistical significance (* *p* < 0.05, ** *p* < 0.01, and *** *p* < 0.001) between the control (Ø AgNP) and AgNP-treated cells.

Analogous to the flow cytometry and qPCR results, THP-1, and diff. THP-1 cells employed similar immunometabolism in response to AgNPs, while coelomocytes’ metabolic response was converse to their human counterparts. AgNPs’ exposure caused impaired mitochondrial respiration in THP-1 and diff. THP-1, which was evidenced by a reduced OCR (Figure 8a,b).

Particle-exposed THP-1 cells also showed decreased respiratory parameters, among which the maximal respiration and spare respiratory capacity significantly differed from the ø AgNP controls. This might indicate the weaker mitochondrial performance of human immunocytes under the stress of AgNP exposure.

However, it might also suggest metabolic reprogramming in human immunocytes during the immune response. The TCA cycle is impaired in proinflammatory macrophages, and leads to fatty acid synthesis, indicating that these cells barely perform oxidative phosphorylation [91]. Notably, synthesized fatty acids are utilized during cell membrane growth, particularly for endocytosis. In any case, the electron transport chain might be disrupted in favor of NO production in macrophages [92], since we also previously reported the AgNPs-induced oxidative stress and ROS induction in both human [5,93] and earthworm [5,79] immunocytes. Analogous changes in the dropped OCR, ECAR, and maximal respiration were observed in response to iron and silica NPs [94,95]. Potentially, the capacity of AgNPs to impair cellular metabolism can be used for the design of therapeutic nanocarriers.

Earthworm coelomocytes showed different responses compared with their human counterparts. Interestingly, unexposed coelomocytes weakly relied on mitochondrial respiration, while AgNPs elevated OCR and significantly increased maximal respiration in coelomocytes (Figure 8c). We also detected AgNP-induced elevation of ECAR, which corresponds to lactate production and is a sign of increased glycolysis. This might be connected to the fact that, during the response to PAMPs, phagocytic cells use glycolysis, which can rapidly ensure intermediates for biosynthesis and support the PPP and TCA cycle [96]. We hypothesized that coelomocytes use both oxidative phosphorylation and glycolysis during the response to AgNPs. Curiously, another hazardous metal in soil, cadmium, did not induce respiratory metabolic changes in earthworms [97].

## 4. Conclusions

Due to the extensive application of AgNPs, their interactions with biological organisms and the related toxicologic consequences deserve special attention from researchers. Previously, we compared the evolutionarily conserved stress responses of *E. andrei* coelomocytes and human THP-1 monocytes evoked by AgNPs’ exposure [5]. Hence, we concluded that earthworm and human immune cells shared functional similarities in the response towards AgNPs.

Here, we aimed to unravel and compare AgNPs’ uptake mechanisms utilized by earthworm and human immunocytes. Our ultrastructural results revealed that all studied cell types engulfed AgNPs and those were not translocated into the nucleus, which might be due to the limited size of nuclear pores to pass the NPs inside, and confirmed the importance of endo/lysosomes in AgNPs intracellular response [98,99]. As for the detailed uptake mechanisms, THP-1 and diff. THP-1 cells maintain particle endocytosis utilizing several pathways simultaneously and/or interchangeably involving SRs, TLR/MyD88 signaling, and microtubules through PI3K, mainly via macropinocytosis. In contrast, earthworm coelomocytes exclusively prefer actin-dependent phagocytosis to endocytose AgNPs.

## Figures and Tables

**Table 1:** List of pharmacological inhibitors of the endocytosis pathways, mechanisms of their actions, and applied concentrations.

Inhibitor	Target Pathway	Mode of Action	Applied Conditions	References
Cytochalasin B	Phagocytosis	F-actin depolymerization	5 µM, 1 h	[22]
Cytochalasin D			5 µM, 1 h	[23]
Colchicine	Pinocytosis	Disrupting microtubules	100 µM, 2 h	[24]
5-(*N*-ethyl-*N*-isopropyl) amiloride (EIPA)	Macropinocytosis	Blocking the Na^+^/H^+^ exchanger	5 µM, 30 min	[25]
Amantadine	Clathrin-mediated endocytosis (CME)	Blocks the clathrin-coated pits	500 µM, 30 min	[26]
Methyl-ß-cyclodextrin (MßCD)	Caveolae-mediated endocytosis (CvME)	Remove cholesterol from membrane	1 mM, 30 min	[27]
Nystatin	CvME	Cholesterol sequestration	54 µM, 30 min	[28]
Polyinosinic acid (Poly(I))	Various pathways (Scavenger receptor (ScR)-mediated)	Binds SR	287.2 µM, 1 h	[29]
Polycytidylic acid (PolyI)	None (Poly(I) antagonist)	Does not bind SR	107.1 µM, 1 h	[30]
Wortmannin	Macropinocytosis	PI3K inhibition	400 nM, 30 min	[31]

**Table 2:** ICP-MS measurements of intracellular silver contents. THP-1, diff. THP-1 cells, and coelomocytes were exposed to EC_20_ concentrations of AgNPs with or without endocytosis inhibitors. Results are shown as percentages of the total Ag added to cell samples and their standard deviations.

% Total Silver Mass in Cells	Ø Inhibitor	Poly(I)	Colchicine	Wortmannin	Cyt. B	Cyt. D
THP-1	19.72 ± 0.48	11.05 ± 7.99	8.94 ± 1.02	10.34 ± 3.39	-	-
diff. THP-1	17.34 ± 10.11	12.51 ± 9.61	11.38 ± 10.55	13.3 ± 2.99	-	-
Coelomocytes	16.22 ± 0.4	-	-	-	16.62 ± 2.2	19.62 ± 0.73

## Data Availability

Not applicable.

## Supplementary Materials

The following supporting information can be downloaded at: https://www.mdpi.com/article/10.3390/nano12162818/s1, Figure S1: Concentration-dependent mortality curves following the incubation with AgNPs; Figure S2: The survival rate of THP-1 cells (**a**), diff. THP-1 cells (**b**), and coelomocytes (**c**) following pharmacological inhibitor exposure; Figure S3: Representative anti-5-mC immunocytochemistry images of THP-1 cells (**a**), diff. THP-1 cells (**b**), and coelomocytes (**c**), Figure S4: Seahorse XF Cell Mito Stress Test Profile with the main mitochondrial respiration parameters; Table S1: Earthworm and human primer sequences used for real-time PCR analysis; Table S2: Physico-chemical parameters of AgNPs during culture conditions.
