# Supplementary material for: Distinct Uptake Routes Participate in Silver Nanoparticle Engulfment by Earthworm and Human Immune Cells
Source: Nanomaterials (Basel). 2022 Aug 17;12(16):2818. doi: 10.3390/nano12162818 (PMC9413649; doi:10.3390/nano12162818)
Supplement: Supplementary file 1 [file nanomaterials-12-02818-s001.zip › nanomaterials-1863492-SI.pdf]

SUPPLEMENTARY MATERIAL

Distinct uptake routes participate in silver nanoparticle engulfment by earthworm and human immune cells

Bohdana Kokhanyuk[1], Viola Bagóné Vántus[2], Balázs Radnai[2], Eszter Vámos[2], Gyula Kajner[3], Gábor Galbács[3], Elek Telek[4], Mária Mészáros[5], Mária A. Deli[5], Péter Németh[1], Péter Engelmann[1,*]

[1]Department of Immunology and Biotechnology, Clinical Center, Medical School, University of Pécs, Pécs, Hungary

[2]Department of Biochemistry and Medical Chemistry, Medical School, University of Pécs, Pécs, Hungary

[3]Department of Inorganic and Analytical Chemistry, Faculty of Science and Informatics, University of Szeged, Szeged, Hungary

[4]Department of Biophysics, Medical School, University of Pécs, Pécs, Hungary

[5]Institute of Biophysics, Biological Research Centre, Eötvös Loránd Research Network, Szeged, Hungary

***Corresponding author:**

Department of Immunology and Biotechnology, Clinical Center, Medical School, University of Pécs, Pécs, H-7643, Szigeti u. 12, Hungary. Tel: + 36-72-536-288, Fax: + 36-72-536-289, email: engelmann.peter@pte.hu

TABLE OF CONTENTS **2**

1. MATERIALS AND METHODS **3**

1.1. Transmission electron microscopy (TEM) **3**

1.2. UV/VIS spectrophotometry, dynamic light scattering (DLS) and zeta potential measurements **3**

1.3. Dose response curve analysis **3**

1.4. RNA-isolation, cDNA synthesis, and real-time PCR **4**

1.5. 5-methylcytosine (5-mC) immunocytochemistry **4**

1.6. Quantification of DNA methylation levels **5**

1.7. Seahorse XF Cell Mito Stress Test **5**

2. REFERENCES………………………………………………………………………...**6**

3. TABLES **7**

4. FIGURES **9**

1. MATERIALS AND METHODS

1.1. Transmission electron microscopy (TEM)

AgNPs (Nanocomposix) were prepared for size and shape observations by transmission electron microscope (TEM). AgNPs from stock solutions were incubated at different exposure conditions and desired concentrations. After washing, the NP pellet was resuspended in 20 µL ddH_2O and further diluted (1:1 or 1:10). The drop of the solution was placed onto the middle of 300 mesh grids coated with colloid ion film (Hatfield, USA, PA) and left to dry for 48 h. The AgNPs were imaged under a JEOL-1400 transmission electron microscope (JEOL Ltd., Tokyo, Japan) and the size distributions of AgNPs were defined.

1.2. UV/VIS spectrophotometry, dynamic light scattering (DLS) and zeta potential measurements

To assess the aggregation state of 75nm AgNPs in different culture conditions, the AgNPs were incubated in ddH_2O, RPMI-1640 with 1% FBS at 37°C or RT and analyzed by UV/VIS spectrophotometry. The light absorbance characteristic of the localized surface plasmon resonance was quantified within the wavelength of 300-800 nm. Samples were diluted in 1:200 and measured with Jasco V-660 UV/VIS Spectrophotometer (JASCO, Tokyo, Japan). The hydrodynamic size and stability of AgNPs were evaluated in different exposure conditions by dynamic light scattering and zeta-potential measurements on a Malvern Zetasizer Nano ZS instrument (equipped with a He-Ne laser (λ = 632.8 nm), Malvern Panalytical Ltd., Cambridge, UK). Means were calculated from the average of at least 3 × 13 measurements per sample.

1.3. Dose-response curve analysis

Target cells were cultured in 24-well plates and exposed to different AgNPs concentrations (0-10 µg/mL for THP-1 and diff. THP-1 cells; 0-100 µg/mL for coelomocytes) for 24 hours at 37°C (THP-1 and diff. THP-1) or RT (coelomocytes). RPMI medium supplemented with 1% heat-inactivated fetal bovine serum and 1% penicillin/streptomycin were applied as a culture medium in all experiments. For cell viability detection, cells were stained with 7-aminoactinomycin D (7-AAD, 1 µg/mL, Biotium, Fremont, CA, USA) and were measured by a FACSCalibur flow cytometer (Beckton Dickinson, Franklin Lakes, NJ, USA) in FL3 (670 LP filter). During flow cytometry, 30,000 events per sample were measured. Using Prism v5.0 (GraphPad Software, La

Jolla, CA, USA) the 4-parameter logistic non-linear regression curve fit was applied based on the results obtained from cell viability assays and EC_{20} values were calculated. The average EC_{20} values obtained from 7-AAD measurements (n=3) were applied in further experiments: 3.1 µg/mL for THP-1 cells, 3.6 µg/mL for diff. THP-1 and 38.9 µg/mL for coelomocytes.

1.4. RNA isolation, cDNA synthesis, and real-time PCR

After RNA isolation and prior to cDNA synthesis, the DNase I digestion (Amplification Grade DNase I; Sigma-Aldrich) was performed using a thermal profile at 25°C for 15 min, then 72°C for 10 min. The cDNA synthesis was executed using the High Capacity cDNA Reverse Transcription Kit (Thermo Fisher Scientific) according to the manufacturer's protocol. cDNAs were stored at -20°C until used as qPCR reaction templates.

The gene-specific primers were designed with Primer Express Software (Thermo Scientific) (please see Table S1) as was described earlier [1]. Target mRNA expressions were measured applying a Maxima SYBR Green Master Mix (Thermo Fisher Scientific) with an ABI Prism 7500 (Applied Biosystems, Waltham, MA, USA). The thermal profile started at 95°C for 10 minutes, followed by 40 cycles of denaturation (35 s at 95°C), hybridization (35 s at 58°C), and elongation (1 min at 72°C) stages with ultimately a dissociation step. The volume of each reaction is 25 µL. Quantitative measurements were normalized to *TATA binding-protein* (*TBP*) for THP-1 and diff. THP-1 or *RPL17* for coelomocytes mRNA level [1]. Four independent experiments were performed.

1.5. 5-methylcytosine (5-mC) immunocytochemistry

Cells were collected and treated as previously described, then cells (80 µL of 1×10^5/mL) were spread onto slides using Cytospin 3 (SHANDON, Thermo Scientific, Waltham, MA, USA) and left to dry overnight. Following day, the samples were fixed in 4% PFA for 20 min, and washed in PBS/0.1% Triton-X 100. Next, slides were incubated with 1 mg/mL phenylhydrazine hydrochloride in PBS/0.1% Triton-X solution for 20 min. After the washing step, 5% BSA in PBS/0.1% Triton-X 100 was applied for 20 min to inhibit the non-specific binding. Samples were incubated with an anti-5-mC monoclonal antibody (Eurogentec, Seraing, Belgium), 1:100 dilution, for 1 h, then horseradish peroxidase (HRP)-linked goat anti-mouse IgG (Dakopatts, Glostrup, Denmark) was added in 1:100 dilution for another 60 min. After washing, 3,3'-

Diaminobenzidine (DAB) substrate was applied for reaction development. Slides were observed with an Olympus BX61 microscope (Olympus Hungary, Budapest), and images were captured by Zeiss Zen software (Carl Zeiss AG, Oberkochen, Germany).

1.6. Quantification of DNA methylation levels

For quantification of 5-methylcytosine (5-mC), 50 ng of genomic DNA (after a renaturation step) was applied onto the nitrocellulose membrane (General Electric Healthcare, Chicago, IL, USA) next to diluted DNA as a standard for quantification. The membranes were dried for 1 h at RT, then for 2 h at 80°C. Then the blocking step using 1% BSA/TBS-T buffer was performed on a shaker for 1 h followed by incubation with anti-5-mC monoclonal antibody (Eurogentec), 1:1000 dilution, for 2 h, RT. Next, membranes were washed 3 times with TBS-T and incubated with horseradish peroxidase (HRP)-conjugated goat anti-mouse IgG (1:1000, Dakopatts). Negative controls (unmethylated CpG, ODN 2006, 50 ng/µL, Hycult Biotech, Wayne, PA, USA), as well as primary and secondary antibodies controls, were also used. For ECL signal detection the SuperSignal Pico solution (Thermo Fisher Scientific) and the ChemiDoc imaging system (Bio-Rad, Hercules, CA, USA) were applied. The dots were quantified using Image Lab 6.0 software (Bio-Rad).

1.7.Seahorse XF Cell Mito Stress Test

Seahorse XF Cell Mito Stress Test (Agilent, Santa Clara, CA, USA) was applied with the following inhibitors at the indicated final concentrations: 1 µM of oligomycin, 1 µM of FCCP, and 1 µM of rotenone–antimycin A. For total cell protein quantification, cells were lysed with RIPA buffer (150 mM NaCl, 50 mM Tris/HCl, 1% (v/v) NP-40, 0.5% (w/v) Na-deoxycholate, 5 mM EDTA, 0.1% SDS, pH 8.0), centrifuged and supernatants were collected. Protein content was quantified by applying the BCA Protein Assay kit (Sigma-Aldrich).

Seahorse XF Cell Mito Stress Test detects parameters of mitochondrial function, such as oxygen consumption rate (OCR) and extracellular acidification rate (ECAR) in real time. At the beginning of the assay, the basal respiration is measured. Later, the modulators of respiration are added into the wells: oligomycin, an inhibitor of ATP synthase, to reveal cellular ATP production; carbonyl cyanide-4 (trifluoromethoxy) phenylhydrazone (FCCP), an uncoupling agent to measure

maximal respiration and spare respiratory capacity; rotenone + antimycin A mixture inhibit complex I and III respectively to calculate non-mitochondrial respiration.

2. REFERENCES

1. Kokhanyuk, B.; Bodó, K.; Sétáló, G.Jr.; Németh, P.; Engelmann, P. Bacterial engulfment mechanism is strongly conserved in evolution between earthworm and human immune cells. *Front. Immunol.*, **2021**, *12*, 733541. https://doi.org/10.3389/fimmu.2021.733541

3. TABLES

Table S1. Earthworm and human primer sequences used for real-time PCR analysis. [a]Upper and lower primer sequences indicate forward and reverse primers.

Species	Target gene	Gene Bank Accession #	Sequence (5'-3')[a]	Amplicon size (bp)
Eisenia andrei	*RPL 17*	BB998250	GCA GAA TTC AAG GGA CTG GA<br>CTC CTT CTC GGA CAG GAT GA	159
	TLR	JX898685	ATT GTG TCA AAC GCC TTC GC<br>GTC GGC GAT CTC TTC CAA CA	123
	MyD88	EH670202	TGC GAG TAC AGG CTC GTT AAC<br>CGT GCA GAT GTG GTT TAG GA	100
	LBP/BPI	JQ407018	GGT TCG ACC TCC GAC GAT AC<br>GGT CAA CAG GGC GTC CAT TA	107
Homo sapiens	*TBP*	BC110341	CCA GAC TGG CAG CAA GAA AAT<br>TCA CAG CTC CCC ACC ATA TTC	100
	TLR	NM_003266	AAA GCC GAA AGG TGA TTG TTG T<br>ACT GCC AGG TCT GAG CAA TCT C	90
	MyD88	NM_0024688	TGA CTT CCA GAC CAA ATT TGC A<br>GAA CTC TTT CTT CAT TGC CTT GTA CT	94
	BPI	NM_001725	TGGCATGCACACAACTGGTT<br>AGTTCCAGGAGCAGCCTATCC	90

Table S2. Physico-chemical parameters of AgNPs during culture conditions. [a,b] Hydrodynamic diameter values (nm) and polydispersity index (PDI) measured by dynamic light scattering are represented as mean ± SD; [c] ζ potential values are present as mean ± SD. All samples were measured in 3 cycles for 13 measurements.

	**Culture medium**	**Mean hydrodynamic diameter (nm)[a]**	**Polydispersity index (PDI)[b]**	**ζ potential (mV)[c]**
**75 nm AgNPs at RT**	RPMI-1640 + 1% FBS	110.60 ± 0.40	0.08 ± 0.02	-9.26 ± 0.91
**75 nm AgNPs at 37°C**	RPMI-1640 + 1% FBS	113.60 ± 1.50	0.10 ± 0.01	-25.30 ± 1.25

4. FIGURES

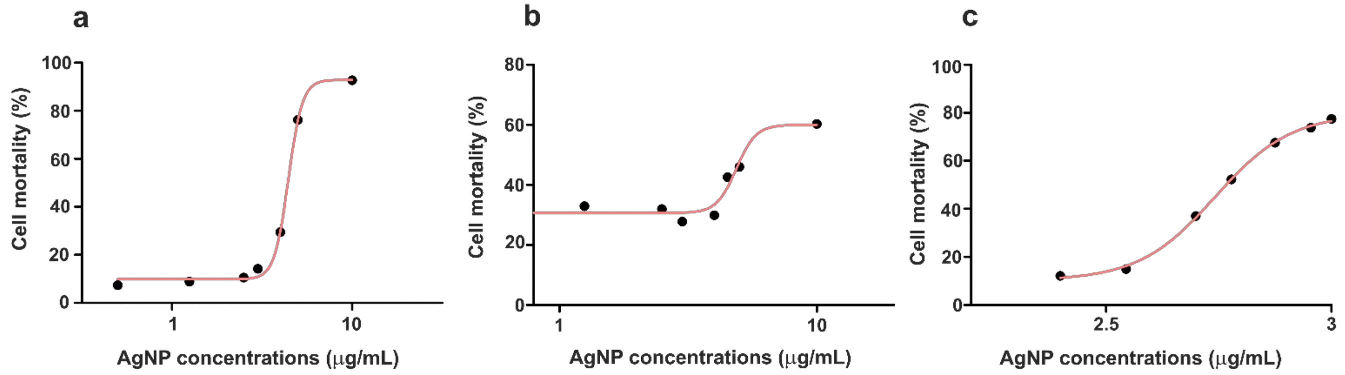

Figure S1. Concentration-dependent mortality curves following the incubation with AgNPs. Mortality of THP-1 (**a**), diff. THP-1 cells (**b**) upon 0-10 µg/mL AgNP and coelomocytes (**c**) upon 0-100 µg/mL AgNPs treatments during 24 h. Data was obtained by flow cytometry after 7-AAD staining. Dots are showing the mean values of three independent measurements.

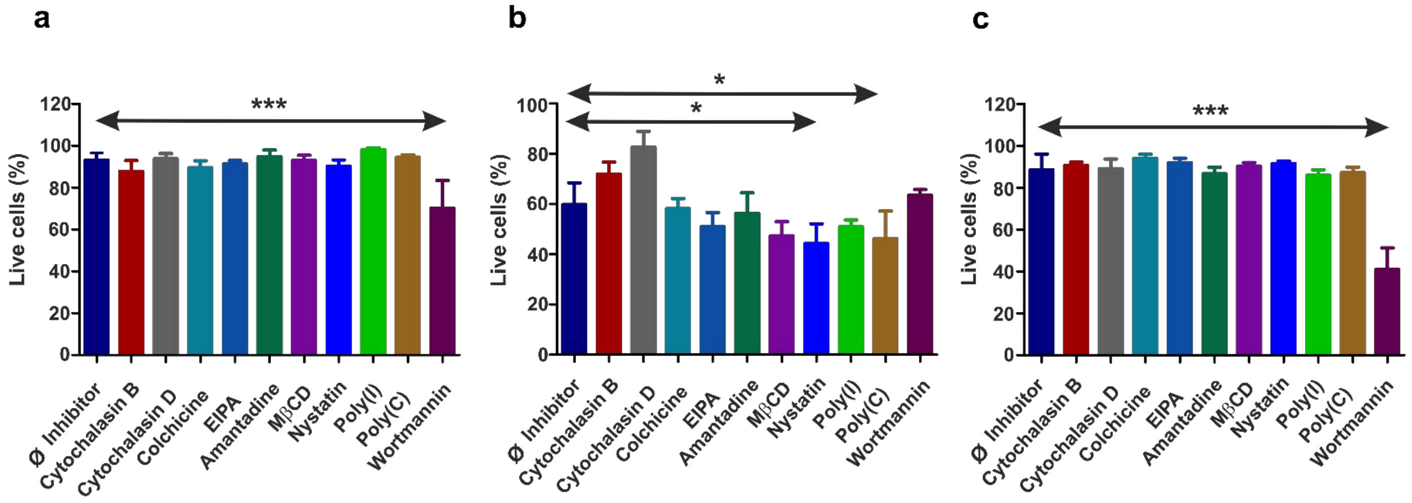

Figure S2. The survival rate of THP-1 cells (**a**), diff. THP-1 cells (**b**) and coelomocytes (**c**) following pharmacological inhibitor exposure. Cytotoxicity of various inhibitors was observed by 7-AAD live/dead cell assay by flow cytometry following 24 h incubation. Results are presented as mean ± SD, n=4. Asterisks signify statistically significant differences (*$p < 0.05$,***$p < 0.001$) between the Ø inhibitor control and various treatments.

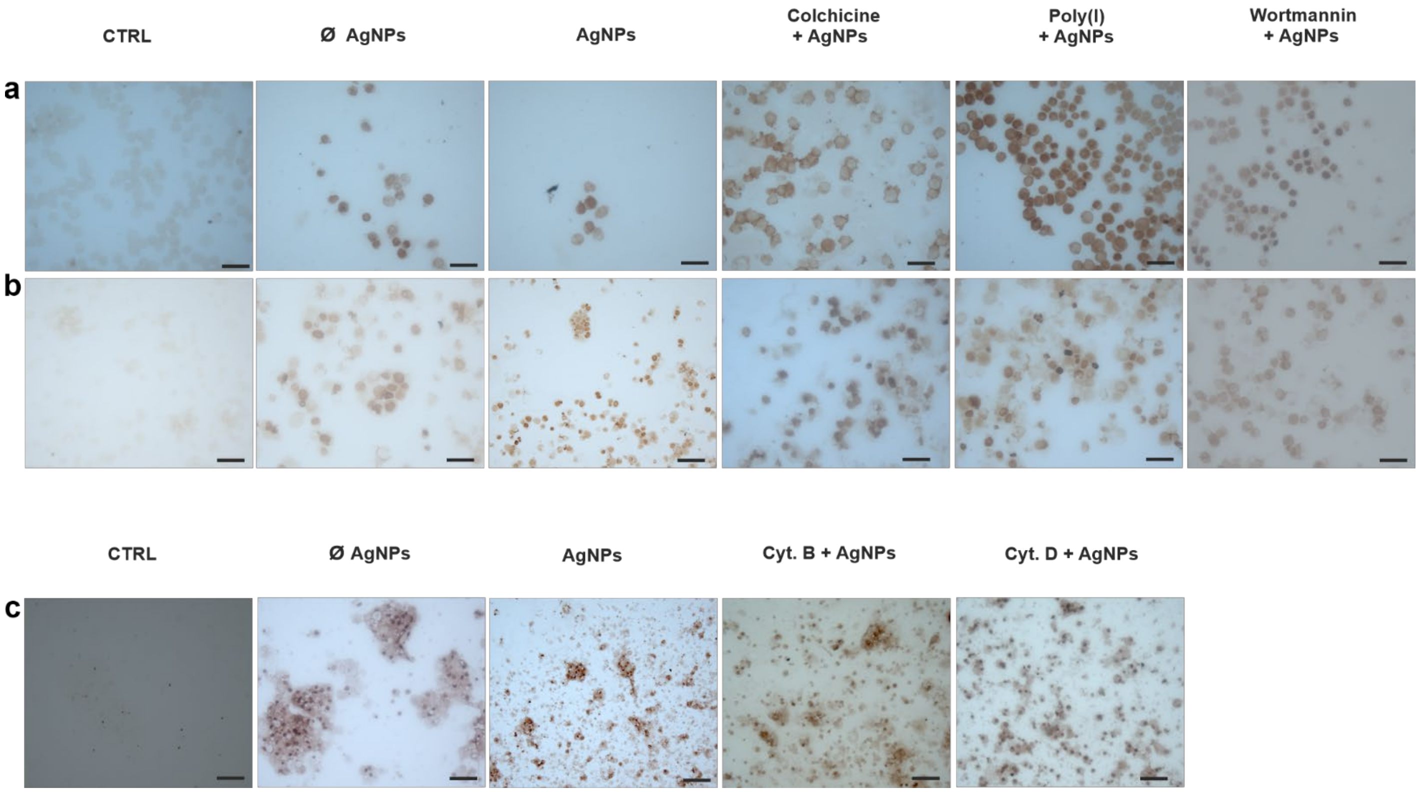

Figure S3. Representative anti-5-mC immunocytochemistry images of THP-1 cells (**a**), diff. THP-1 cells (**b**) and coelomocytes (**c**). Note the differences of CTRL samples (anti-5-mC antibody omitted) compared to target cells exposed to different conditions (incubated with anti-5-mC monoclonal antibody). Scale bars: 200 µm.

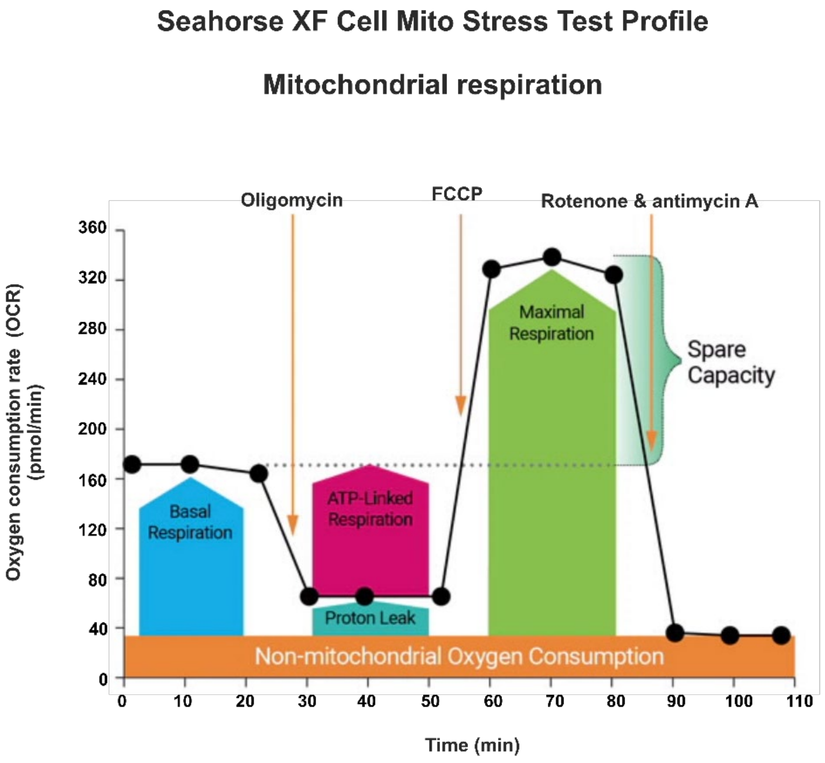

Fig. S4. Seahorse XF Cell Mito Stress Test Profile with the main mitochondrial respiration parameters (Agilent Technologies, Inc.).
